# Psychometric properties of the Work Limitations Questionnaire applied to nursing workers*

**DOI:** 10.1590/1518-8345.4771.3466

**Published:** 2021-08-30

**Authors:** Samuel Andrade de Oliveira, Juliana Alvares Duarte Bonini Campos, João Marôco, Maria Helena Palucci Marziale, Fernanda Ludmilla Rossi Rocha

**Affiliations:** 1 Universidade de São Paulo, Escola de Enfermagem de Ribeirão Preto, PAHO/WHO Collaborating Centre for Nursing Research Development, Ribeirão Preto, SP, Brazil.; 2 Scholarship holder at the Coordenação de Aperfeiçoamento de Pessoal de Nível Superior (CAPES), Brazil.; 3 Universidade Estadual Paulista, Faculdade de Ciências Farmacêuticas, Araraquara, SP, Brazil.; 4 Instituto Universitário de Ciências Psicológicas, Sociais e da Vida, Lisboa, PT, Portugal.

**Keywords:** Nursing, Occupational Health, Workers, Presenteeism, Psychometrics, Validation Studies, Enfermagem, Saúde do Trabalhador, Trabalhadores, Presenteísmo, Psicometria, Estudos de Validação, Enfermería, Salud Laboral, Trabajadores, Presentismo, Psicometría, Estudios de Validación

## Abstract

**Objective:**

to evaluate the psychometric properties of the Work Limitations Questionnaire and to measure presenteeism in a sample of nursing workers.

**Method:**

a cross-sectional study, with non-probabilistic sampling. Data was collected between July 2018 and February 2019 in two high-complexity hospitals, and the sample was composed of 304 participants. The validity analysis of the Work Limitations Questionnaire was performed by means of Confirmatory Factor Analysis.

**Results:**

most of the participants were women (88.5%), with a mean age of 40.9 years old. The validities of the factorial, convergent and discriminant construct and the reliability of the complete version and of the 16-item version of the instrument were adequate after adjusting the models to the sample. A statistically significant and negative correlation (p<0.001) was verified between the workload, working time and the Time Management and Physical Demands dimensions; as well as a statistically significant (p<0.001) correlation between working time and the Mental-Interpersonal Demands and Production Demands dimensions. Gender and professional category did not influence presenteeism. The rate of loss of productivity at work was 19.51%.

**Conclusion:**

the Work Limitations Questionnaire showed adequate validity and reliability and can be considered a valid and reliable instrument for assessing presenteeism in the nursing team.

## Abstract (pt)

**Objetivo:**

avaliar as propriedades psicométricas do *Work Limitations Questionnaire* e mensurar o presenteísmo em uma amostra de trabalhadores de enfermagem.

**Método:**

estudo transversal, com amostragem não probabilística. Os dados foram coletados entre julho de 2018 e fevereiro de 2019 em dois hospitais de alta complexidade e a amostra foi composta por 304 participantes. A análise da validade do *Work Limitations Questionnaire* foi realizada por meio da Análise Fatorial Confirmatória.

**Resultados:**

a maioria dos participantes eram mulheres (88,5%), com média de idade de 40,9 anos. As validades de construto fatorial, convergente e discriminante e a confiabilidade da versão completa e da versão de 16 itens do instrumento foram adequadas após ajustamento dos modelos. Verificou-se correlação estatisticamente significativa e negativa (p<0,001) entre carga horária, tempo de trabalho e as dimensões Gerência de Tempo e Demandas Físicas; correlação estatisticamente significativa (p<0,001) entre tempo de trabalho e as dimensões Demandas Mentais-Interpessoais e Demandas de Produção. Sexo e categoria profissional não influenciaram o presenteísmo. O índice de perda de produtividade no trabalho foi de 19,51%.

**Conclusão:**

o *Work Limitations Questionnaire* apresentou adequada validade e confiabilidade e pode ser considerado um instrumento válido e confiável para avaliação do presenteísmo na equipe de enfermagem.

## Abstract (es)

**Objetivo:**

evaluar las propiedades psicométricas del *Work Limitations Questionnaire* y medir el presentismo en una muestra de trabajadores de enfermería.

**Método:**

se trata de un estudio transversal, con muestreo no probabilístico. Los datos se recolectaron entre julio de 2018 y febrero de 2019 en dos hospitales de alta complejidad y la muestra estuvo compuesta por 304 participantes. El análisis de validez del *Work Limitations Questionnaire* se realizó mediante Análisis Factorial Confirmatorio.

**Resultados:**

la mayoría de los participantes era mujer (88,5%), con una edad media de 40,9 años. La validez del constructo factorial, convergente y discriminante, y la confiabilidad de la versión completa y de la versión de 16 ítems del instrumento fueron adecuadas luego de ajustar los modelos. Hubo una correlación estadísticamente significativa y negativa (p <0,001) entre carga horaria de trabajo, tiempo de trabajo y las dimensiones Gestión del Tiempo y Demandas Físicas; correlación estadísticamente significativa (p <0,001) entre el tiempo de trabajo y las dimensiones Demandas Mentales Interpersonales y Demandas de Producción. El sexo y la categoría profesional no influyeron en el presentismo. La tasa de pérdida de productividad en el trabajo fue del 19,51%.

**Conclusión:**

el *Work Limitations Questionnaire* presentó adecuada validez y confiabilidad y puede ser considerado un instrumento válido y confiable para evaluar el presentismo en el equipo de enfermería.

## Introduction

In the current context of the world of work, presenteeism emerges as an increasingly growing phenomenon, which occurs when the individual is physically present in the workplace, but functionally absent, due to health problems^(1)^. As a consequence, the presence of the sick worker at work causes reduced productivity and may come to aggravate existing health problems, compromising the worker’s quality of life^(2)^.

Globally, presenteeism has become a concern, as it can generate higher costs than absenteeism for the institutions. For this reason, its prevalence has been studied in countries such as USA, UK, Canada, Denmark, Sweden, Finland, Germany, Spain, Italy, Taiwan, South Korea, Sri Lanka and Saudi Arabia, reaching rates of presenteeism that vary from 30% to 90%^(1)^.

Among the workers who are prone to presenteeism, nursing is one of the professions with a high risk of exhaustion, stress and illness due to numerous occupational factors such as, for example, the inadequate number of professionals in the institutions, which causes work overload and possibility of physical and mental illness in the individuals, favoring the occurrence of presenteeism^(3)^. A number of studies show that presenteeism is common among nurses and is related to reduced quality of care and lower organizational efficiency^(4-8)^.

Among the nursing workers, the causes of presenteeism can also be related to the organizational culture and to work organization, one of the main factors being the pressure from managers and the relationships with coworkers^(4-6)^. In addition to organizational factors, individual aspects are directly related to presenteeism, as is the case with the physical and psychological conditions of the nursing workers, who are often affected by musculoskeletal disorders and problems such as anxiety and depression related to work stress^(3,5)^.

Given its complexity, it becomes difficult to measure presenteeism, which is why it is not yet possible to assert with certainty what its real consequences are for the workers’ health and for the organizations. In recent decades, however, different tools for evaluating presenteeism have been developed in order to quantify health-related productivity changes^(1,7)^.

Of the instruments used worldwide to assess presenteeism, the following were adapted and applied to the Brazilian context: Stanford Presenteeism Scale (SPS6)^(8)^; Health and Work Performance Questionnaire (HPQ)^(9)^; Work Limitations Questionnaire (WLQ)^(10)^; and Work Productivity and Activity Impairment (WPAI)^(11)^.

Among these instruments, the WLQ is internationally recognized and has been translated into more than 40 languages and culturally adapted in several countries, such as Brazil(10,12), Portugal(13), Japan(14), USA(15-17), Canada^(18-19)^ and Holland^(20)^. The WLQ represents an instrument that measures the degree of interference of health problems in the ability to perform tasks at work and in the individual’s productivity and, together, its dimensions encompass the multidimensional character of the functions developed at work and can elucidate in which domains the individual has limited functions^(21)^. These factors were decisive for selecting the WLQ as the instrument to be used in this research.

The WLQ was prepared being based on the disability framework and the assumptions of the Demand-Control Model^(22)^. Disability is the result of a complex interaction between a person’s functional limitations (health-related restrictions on the ability to perform tasks and social role obligations) and the physical and social environment in which such performance occurs^(23)^. When measuring to what extent health problems interfere with an individual’s ability to perform their duties at work, the WLQ indicates how much an inability to perform tasks at work can generate consequences both individually and in the worker’s social context^(21)^. This reflects the theoretical model of disability which shows that a limitation goes far beyond physical issues, but is a relationship of the biopsychosocial process in the context in which each individual is inserted^(24)^.

The Demand-Control Model^(22)^ is structured by two dimensions: demand and control. Work demands represent the physical and psychological demands inherent to the activities carried out in work environments, while control represents the worker’s autonomy over work to make decisions and develop skills. According to this model, the greater the work demands and the lesser the individuals’ control over their activities, the greater the physical and/or psychological exhaustion of workers, which can lead to work-related illness^(22)^. In this sense, the WLQ allows identifying the main work demands that may come to affect the workers’ health and productivity at work.

In this context, the objective of the study herein presented was to evaluate the psychometric properties of the adapted version for Brazil of the Work Limitations Questionnaire^(10)^ and to measure presenteeism in a sample of nursing workers.

## Method

### Study design, locus, population and sample

This is a cross-sectional and observational study, with non-probabilistic sampling, carried out in two high-complexity general public hospitals, one located in the Northern region of the state of Paraná and the other in the Northwest region of the state of São Paulo, both in municipalities considered references for health treatment and with high population density.

A total of 600 nurses, nursing technicians and assistants working in the aforementioned institutions were invited to participate in the research. The inclusion criteria considered were the following: working for at least six months in the institutions; not being away from work during the data collection period; referring having health problems; and reporting having worked ill in the last two weeks prior to the date of data collection.

The established criteria are based on the theoretical assumptions that supported the elaboration of the WLQ, which consider presenteeism as a phenomenon resulting from health problems that interfere in the individual’s working capacity^(21,23)^.

The minimum sample size was estimated considering the need for five to 10 respondents *per* parameter to be estimated^(25)^. As the WLQ has 25 items, 56 parameters were considered (25 items, 25 errors and six correlations between the factors), requiring a minimum sample size of 280 to 560.

A total of 476 workers (adherence rate = 78.6%) agreed to participate in this study. However, according to the inclusion criteria adopted, 172 (36.1%) workers denied having any health problem and having worked ill in the last two weeks, being excluded from the sample. Thus, the sample was composed of 304 participants.

### Data collection

Data collection took place between July 2018 and February 2019. Nursing workers active in all sectors of the two institutions studied in this research were personally invited to participate. The invitation was made individually and during working hours, and up to three attempts were made to approach each worker. All the participants received information about the study and signed an Informed Consent Form (ICF).

After signing the informed consent form, the participants were invited to go to a private room and, in the presence of the researcher, but without interference, they answered the data collection instruments. Everyone had the option to fill out the forms immediately or to answer them at a time they deemed most appropriate; in these cases, a new period for the collection of information was scheduled.

### Data collection instruments

To characterize the participants, an instrument was used with demographic and occupational information such as age, gender, marital status, schooling level, professional category, working time in nursing and in the institution, weekly workload, type of employment contract, presence/absence of double employment contract and health conditions of the workers, which was subjected to a validation process by the Expert Committee before being applied^(26)^.

Regarding the type of employment contract, the following categories were considered: civil servants (represented by the workers who passed public tenders and who become effective collaborators of governmental entities); employees working under the Labor Laws Consolidation (*Consolidação das Leis de Trabalho*, CLT) (represented by the workers whose employment contracts are governed by the CLT and whose individual or collective work rules are determined by entities representing the different professional categories); call, which represents a specific contract from one of the hospitals under study and refers to a type of individual contract for the provision of services in the form of duty at the institution.

To evaluate presenteeism, the Work Limitations Questionnaire (WLQ) was used, an instrument originally developed by Lerner and collaborators^(21)^ in the English language. The WLQ is a self-applicable tool, which asks the participant to assess their own degree of difficulty to perform specific tasks required in their work. It has 25 items arranged in four domains: Time Management – TM (five items), Physical Demands – PD (six items), Mental-Interpersonal Demands – MID (nine items) and Output Demands – OD (five items). The answers to the items are arranged on a 5-point Likert scale, varying from 0 (without limitation) to 100 (all the time with limitation)^(10,21)^.

The Physical Demands (PD) domain has an inverted statement in relation to the others; in view of this, the authors of the original version of the instrument advise that items belonging to the TM, MID and OD domains have their answer scales inverted. To obtain the final WLQ score, there is a manual with guidelines through which it is possible to calculate the global WLQ score and the index of lost productivity at work^(16)^.

In addition to the original version with 25 items, using the same theoretical bases as the original version, reduced versions of WLQ have also been proposed, such as WLQ-16^(27)^ and WLQ-8^(28-30)^. Initially, the 16-item version was developed for a study of carpal tunnel syndrome and maintained the four domains of the original version^(27)^. The eight-item version was developed based on the eight issues predictive of economic results related to the loss of productivity of the original version^(13)^. Given its short size, WLQ-8 is commonly used in non-research settings, as a tool for rapid assessment of workers’ health^(28-30)^.

In Brazil, the WLQ was translated and culturally adapted for the first time in 2007^(10)^, being the version used in this study.

It is noted that, for the use of WLQ, authorization was obtained from the *Mapi Research Trust,* a non-profit organization coordinated by the authors of the original version of the instrument.

### Data analysis

Descriptive statistics was used to analyze the data related to the characterization of the participants. To validate the WLQ, the psychometric sensitivity of the items, the validity of the factorial, convergent and discriminant construct, the validity of divergent and competing criteria, and the reliability of the instrument were estimated.

The psychometric sensitivity of the WLQ items was assessed using summary (mean, median and standard deviation) and form (skewness and kurtosis) measures of the participants’ answers, being considered adequate when the absolute values of skewness and kurtosis were below three and seven, respectively, that is, when the distribution of the items did not severely violate normal distribution^(31-32)^.

The factorial construct validity was tested by means of Confirmatory Factor Analysis (CFA), using the maximum likelihood estimation method. To assess the quality of fit of the models to the data, the ratio of chi-square and degrees of freedom (*x* ^2^/df), the Comparative Fit Index (CFI), the Tucker-Lewis Index (TLI) and the Root Mean Square Error of Approximation (RMSEA) were used, the following being considered adequate values: *x* ^2^/ df≤ 5.0; CFI and TLI ≥ 0.90; and RMSEA ≤ 0.08^(32-34)^. In addition, the factorial weights (λ) and modification indexes were calculated using the Lagrange multipliers (LM), with λ ≥ 0.50 being considered adequate and the trajectories and/or correlations with LM >11 being analyzed^(25,32)^.

The convergent construct validity of the WLQ domains was assessed based on the Average Variance Extracted (AVE), considered adequate if AVE ≥ 0.50, and discriminant construct validity was confirmed if AVE_i_ and AVEj ≥ *ρ*ij2^(35)^.

The instrument’s reliability was assessed by means of standardized Cronbach’s alpha coefficient (α) and by Composite Reliability (CR), with α and CC values ≥ 0.70 being considered adequate^(32)^.

The convergent and divergent criterion validity analysis of the WLQ was performed by comparing the scores for each domain of the WLQ according to demographic variables (gender, professional category, weekly workload, and working time in nursing). The correlation between the scores of each of the WLQ domains, workload and working time in nursing was estimated using Pearson’s correlation coefficient (r), with a 5% significance level. To compare the scores according to gender and professional category, analysis of variance (ANOVA) was performed, separately for each variable. The assumptions of normality and homoscedasticity of the data (Levene’s test) were evaluated and Welch’s correction was used in cases of rejection of the assumption of homoscedasticity.

All the statistical analyses were performed using the IBM SPSS Statistics 22 (IBM Corp., Armonk, N.Y., USA) and AMOS 22.0 (IBM Corp., Armonk, N.Y., USA) programs.

### Presenteeism scores in the sample

After adjusting the complete WLQ model to the data, the instrument’s global score was measured in order to assess the levels of presenteeism in the sample. For this, the guidelines by the authors of the original instrument were followed^(21)^, with three stages being carried out. In stage 1, the arithmetic means of the WLQ domains were calculated, following Equation 1.

(1) $$\text{[WLQScale Score}=25^{*}\text{(mean items scale score-1]}$$

Subsequently, the global WLQ Index was estimated, using Equation 2.

(2) $$\left[\text{WLQ Index}=\left(\beta_{1}\text{WLQ}TM+\beta_{2}\cdot \text{WLQ}PD+\beta_{3}\cdot \text{WLQ}MID+\beta_{4}\cdot \text{WLQ OD}\right)\right]$$

Note: WLQ TM: Time Management; WLQ PD: Physical Demands; WLQ MID: Mental-Interpersonal Demands; WLQ OD: Output Demands

Where: β_1_ = 0.00048, β_2_ = 0.00036, β_3_ = 0.00096, and β_4_ = 000106

In the last stage, the At-Work Productivity Loss Index (Equation 3) was calculated.

(3) [WLQ At-Work Productivity Loss Index = (1-exp (- WLQ Index)]

### Ethical aspects

This study was approved by the Research Ethics Committees of the Universities linked to the hospitals under study (CAAE: 89678518.9.0000.5393 and CAAE: 89678518.9.3001.5231). The recommendations of Resolution 466/2012 of the Ministry of Health were followed, respecting the ethical precepts established on the guidelines and standards of research involving human beings.

## Results

Of the 304 participants, it was verified that the majority were female (88.5%) and that the mean age in the sample was 40.9 (standard deviation (SD)=10.0). It was verified that the mean working time in nursing was 15.9 (SD=9.7) years and that the mean working time in hospitals was 10.5 (SD=9.5) years. As for the working day, 57.9% worked 30 or 36 hours and 82.6% did not have double employment contracts. Most of the workers, 191 (62.8%), were employed as civil servants and 239 (78.6%) were nursing technicians or assistants.

Data related to health conditions showed that 52.6% (n=160) of the workers considered their health status to be good; 33.6% (n=102) judged their health in a regular state; 6.3% (n=19) considered their health to be poor or very bad; and 7.6% (n=23) admitted to having a very good health status. It was verified that, in the last 12 months, 23.7% (n=72) were absent from work due to any health problem and that 63.8% (n=194) of the workers had some health problem, with musculoskeletal diseases in general being the most frequent health problem among the participants, affecting 47.7% (n=145) of the workers. Specifically, it was observed that 28.6% (n=87) of the participants reported being affected by low back pain. In addition to musculoskeletal disorders, stress was reported by 38.8% (n=118) of the workers, anxiety was reported by 35.9% (n=109) of the participants, and respiratory problems were reported by 26.6% (n=81) of the sample. It is worth mentioning that 12.2% (n=37) of the participants reported depression.

Regarding the psychometric properties of the WLQ, the analysis of the psychometric sensitivity of the items showed that the absolute values of skewness were below three (sk=0.56-1.81) and kurtosis below seven (ku=1.433.24), proving the normal distribution of the answers to the items.

**Figure 1:**
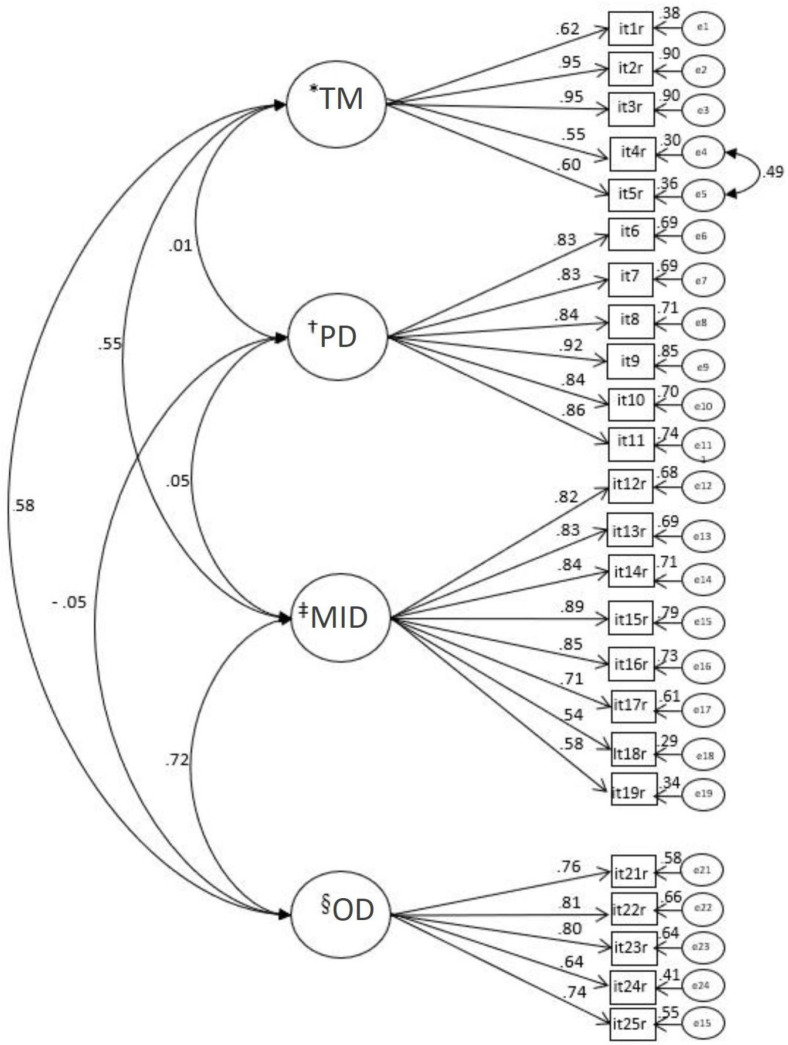
Complete factorial model of the Work Limitations Questionnaire adjusted for the sample of nursing workers (n=304) ^*^TM = Time Management;^†^PD = Physical Demands;^‡^MID = Mental-Interpersonal Demands;^§^OD = Output Demands

The factorial model of the WLQ showed an acceptable fit to the data (*x* ^2^/df =3.51; CFI=0.89; TLI=0.87; RMSEA=0.09). However, a low factor weight of item 20 (λ=0.46) was observed, belonging to the MentalInterpersonal Demands domain, opting for excluding this item. From the Lagrange multipliers, a strong correlation between the errors of items 4 and 5 (LM=71.70) was verified and, therefore, the correlation between these errors was inserted, which resulted in a better adjustment of the model to the data (*x* ^2^/df=2.79; CFI=0.92; TLI=0.91; RMSEA=0.08) (Figure 1).

The adjusted model showed a strong correlation between the MID and OD domains [r=0.72], a moderate correlation between the TM and PD domains [r=0.58] and between TM and MDI [r=0.55] and very weak correlations between the PD domain and the other WLQ domains [r(PDxMID)=0.05; r(PDxTM)=0.01; r(PDxOD)=-0.05].

The validity of the convergent [AVE_PD_=0,73; AVE_MID_=0.59; AVE_(TM)_=0.57; AVE_(OD)_=0.57] and discriminant construct [AVE_(PD)_ and AVE_(TM)_ (r^2^=0.00); AVE_(PD)_ and AVE(MID) (r2=0.00); AVE(PD) and AVE(OD) (r2=0.00); AVE(MID) and AVE_(OD)_ (r^2^=0.52); AVE_(TM)_ and AVE_(OD)_ (r^2^=0.34); AVE_(MID)_ and AVE_(TM)_ (r^2^_=_0.30)] was adequate for all WLQ domains. Reliability was also adequate [α: PD=0.94; MID=0.92; TM=0.88; OD=0.86; CR: PD=0.92; MID=0.87; TM=0.79; OD=0.79].

**Figure 2:**
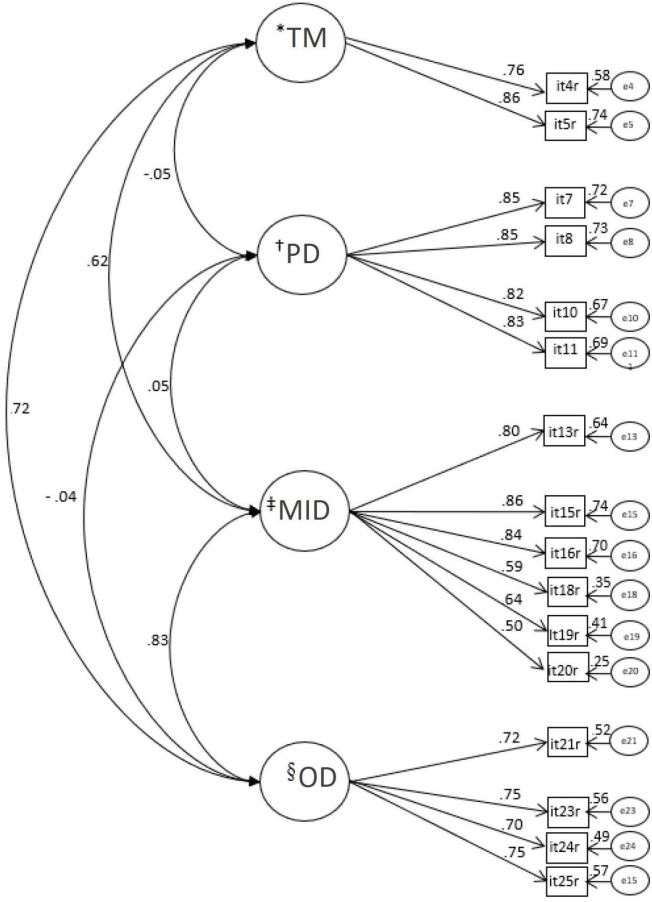
Model of the Work Limitations Questionnaire – 16 items adjusted for the sample of nursing workers (n=304) ^*^TM = Time Management;^†^PD = Physical Demands;^‡^MID = Mental-Interpersonal Demands;^§^OD = Output Demands

Regarding WLQ-16, it was verified that the model also presented an acceptable fit to the data (*x* ^2^/df=3.45; CFI=0.91; TLI=0.89; RMSEA=0.09) (Figure 2).

This factorial model showed strong correlations between the MID and OD domains [r=0.83] and between TM and OD [r=0.72]; moderate correlation between the TM and MDI domains [r=0.62]; and very weak correlations between the PD domain and the other WLQ domains [r(PDxMID)=0.05; r(PDxTM)=-0.05; r(PDxOD)=-0.04]. Adequate convergent validity was found [AVE: PD=0.70; TM=0.66; OD=0.53; MID=0.52], as well as adequate discriminant validity between the domains [AVE(PD) and AVE(TM) (r^2^=0.00); AVE(PD) and AVE(MID) (r^2^=0.00); AVE(PD) and AVE(OD) (r^2^=0.00); AVE(TM) and AVE(OD) (r^2^=0.51); AVE(MID) and AVE(TM) (r^2^=0.39)], with the exception of the MID and OD domains [AVE(MID) and AVE(OD) (r^2^=0.69)], being explained by the high correlation between them. The reliability of the domains was also adequate [α: PD=0.90; MID=0.86; OD=0.81; TM=0.79; CR: PD=0.90; MID=0.86; OD=0.82; TM=0.80].

The adjustment of the factorial model of the reduced version (WLQ-8) for the sample was not possible, since there was no convergence of the data covariance matrix.

After data validation, the WLQ scores were calculated for the sample (Table 1).

**Table 1:** Scores for the domains and global score of the Work Limitations Questionnaire for the sample. Londrina/PR and Ribeirão Preto/SP, Brazil, 2019

WLQ*	**WLQ Domain^†^**	**WLQ Index^‡^**	**WLQ Productivity^§^**
TM^||^	78.08		
PD^¶^	40.28	0.20	19.51%
MID**	80.90		
OD^††^	82.83		

* WLQ = Work Limitations Questionnaire;^†^WLQ Domain = WLQ Scale Score;^‡^WLQ Index = WLQ Index;^§^WLQ Productivity= WLQ At-Work Productivity Loss Index;^||^TM = Time management;^¶^PD = Physical demands; **MID = Mental-Interpersonal Demands;^††^OD = Output Demands

A considerable loss of productivity was observed among the nursing workers, mainly related to the Output Demands, Mental-Interpersonal Demands and Time Management domains, with less contribution from the Physical Demands domain.

**Table 2:** Mean global scores (mean±standard-deviation) for the domains of the Work Limitations Questionnaire according to gender and professional category. Londrina/PR and Ribeirão Preto/SP, Brazil, 2019

Variable	WLQ Dimensions^¶^			
	^||^TM^*^	^||^PD^†^	^||^MID^‡^	^||^OD^§^
**Gender**	(mean ± standard deviation)			
Male (n=35)	3.33 ± 0.96	2.82 ± 1.14	4.46 ± 0.69	4.38 ± 0.69
Female (n=269)	3.32 ± 0.86	2.83 ± 1.12	4.27 ± 0.72	4.29 ± 0.71
Total (n=304)	3.32 ± 0.87	2.83 ± 1.12	4.30 ± 0.72	4.30 ± 0.71
F	0.00	0.00	2.05	0.43
*P*	0.95	0.95	0.15	0.51
Position				
Nurse (n=65)	3.34 ± 0.84	2.90 ± 1.12	4.47 ± 0.63	4.36 ± 0.67
Technician (n=166)	3.25 ± 0.86	2.71 ± 1.06	4.28 ± 0.74	4.33 ± 0.72
Assistant (n=73)	3.47 ± 0.89	3.05 ± 1.21	4.17 ± 0.70	4.18 ± 0.71
Total (n=304)	3.32 ± 0.87	2.83 ± 1.12	4.30 ± 0.72	4.30 ± 0.71
F	1.57	2.66	3.10	1.54
*P*	0.21	0.07	0.05	0.22

^*^TM = Time Management; ^†^PD = Physical Demands; ^‡^MID = Mental-Interpersonal Demands; ^§^OD = Output Demands; ^||^Welch’s Correction; ^¶^WLQ = Work Limitations Questionnaire

**Table 3:** Pearson’s Correlation Matrix between the working time in nursing and weekly workload variables and the domains of the Work Limitations Questionnaire. Londrina/PR and Ribeirão Preto/SP, Brazil, 2019

Variable	Time	Workload	TM*	**PD^†^**	**MID^‡^**	**OD^§^**
^||^Time	1					
Workload	-0.19^**^	1				
TM*	-0.06	-0.18^**^	1			
PD^†^	-0.08	-0.17^**^	0.80^**^	1		
MID^‡^	0.17^**^	-.006	0.35^**^	0.13^¶^	1	
OD^§^	0.15^**^	-0.04	0.32^**^	0.01	0.79^**^	1

^*^TM = Time Management; ^†^PD = Physical Demands; ^‡^MID = Mental-Interpersonal Demands; ^§^OD = Output Demands. ^||^Time = Working time in nursing; ^¶^The correlation is significant at the 0.05 level; ^**^The correlation is significant at the 0.01 level

Table 2 shows the comparison of the global scores for each domain of the WLQ according to the participants’ gender and professional category.

Table 2 shows that no statistically significant differences were observed between the WLQ scores according to gender and professional category.

Table 3 shows the correlation matrix between the domains of the WLQ and the working time in nursing and the workload.

A statistically and negatively significant correlation (p<0.001) was verified between the weekly workload and the working time in the institution and between the workload and the TM and PD dimensions (divergent criterion validity). Therefore, the greater the workers’ workload, the lower the working time in the institution, the physical demands and the time management. In addition, a positive and statistically significant correlation (p<0.001) was found between working time and the MID and OD dimensions (convergent criterion validity).

## Discussion

The evaluation of the psychometric properties of the WLQ for the sample of nursing workers, carried out by means of Confirmatory Factor Analysis, showed adequate validity and reliability of the instrument for the studied context.

Regarding the factorial validity of the WLQ, it was observed that the adjusted model presented adequate goodness of fit indexes, four factors and 24 items, adequate factor weights, moderate and strong correlations between the MID, OD and TM factors and weak correlations between the PD factor and the other instrument-related factors.

Regarding the weak correlations between the Physical Demands domain and the instrument’s domains, these results corroborate the findings of other validation studies of the WLQ^(19,36-38)^, in which strong correlations were also observed between the Output Demands, MentalInterpersonal Demands, and Time Management domains, and weak correlations between such domains and the Physical Demands domain.

A study carried out to estimate the psychometric properties of the WLQ^(23)^ among patients with cancer also stands out, where a similar adjustment of the instrument’s factorial model was observed, with weak correlations involving the Physical Demands domain.

This fact can be related to the inversion of the answer pattern of the items in this domain, formulated in the opposite direction to the other WLQ domains, which compromises the interpretation of the respondent, who does not notice the change and maintains the previous pattern for the answers^(19)^.

Seeking to prove that the cause of this event is related to the inversion of the answer pattern of the items in the Physical Demands domain, a WLQ validation study in workers with rheumatoid arthritis^(39)^ proposed to modify the wording of the items in this domain, following the same answer pattern as the rest of the instrument. After this procedure, high correlations were observed among all domains of the instrument, which proves the importance of the domain for the evaluation of presenteeism and, at the same time, reinforces the problem related to the construction of instruments with inverted answer scales.

Another procedure performed in relation to the Physical Demands was the exclusion of this domain in a WLQ validation study among patients with chronic diseases in the upper limbs^(19)^. The factor analysis of the three-factor model, without the items belonging to the Physical Demands domain, proved the adequate adjustment for the sample. However, the authors stressed out that significantly smaller than expected interfactor correlations involving the Physical Demands domain can have serious implications for future studies.

Considering the specificities of the nursing work, it has been observed that physical health problems, such as musculoskeletal disorders and, in particular, low back pain, have represented one of the main diseases related to the work of these professionals^(3,5)^, which was also verified in this study from the participants’ reports. In addition to that, the theoretical assumptions that underlie the concept of presenteeism consider physical demands as extremely relevant in the process of weariness and illness of the workers^(40-41)^.

Thus, it is considered fundamental to understand the relevant role of the physical demands in the process of wear out and illness of the nursing workers. Based on these aspects, in the study herein presented, it was decided not to exclude the items or the Physical Demands domain during the adjustment of the model for the sample, despite the weak correlation with the other domains of the WLQ, as it is understood that physical health problems are directly related to the presenteeism of nursing workers, causing serious consequences for individuals and organizations, such as worsening health status, exhaustion and reduced productivity^(3,22)^.

In addition to the physical demands, nursing workers face excessive workloads, unhealthy work environments, daily exposure to physical, biological and chemical risks arising from their work activities, factors that can enhance the development and aggravation of physical and psychological diseases in these individuals and contribute to the occurrence of presenteeism^(42)^.

Unlike the Physical Demands domain, the MentalInterpersonal Demands domain showed a strong correlation with the Output Demands domain and a moderate correlation with the Time Management domain. This fact corroborates the assumptions of the DemandControl Model^(22)^, relating inappropriate work processes to the generation of demands of various natures, which lead to psychological wear out and other health problems in the workers.

A study carried out with nursing workers at an Intensive Care Unit showed that high levels of pressure at work and requirements for compliance with rules and routines, the permanent surveillance of supervisors, the inadequate relationship between managers and the team of nursing technicians and assistants and the feeling related to the lack of appreciation of workers in the work environment represented determining factors for the individuals’ psychological suffering, contributing to the occurrence of presenteeism^(43)^.

Regarding the analysis of the convergent construct validity, it was observed that the AVE was adequate for all domains of the WLQ, proving that the items that make up each domain present correlations between them and represent the respective domains^(32)^. The discriminant construct validity was also shown to be adequate for all domains of the instrument, which results from the low correlation of the Physical Demands domain and the other domains of the instrument and the absence of a strong correlation between the domains, demonstrating that the items that reflect a domain are not strongly correlated to another domain^(32)^. In this way, the accuracy of the Work Limitations Questionnaire was demonstrated in the sample of nursing workers.

The analysis of the internal consistency of the data revealed adequate CR and α values, corroborating results from other validation studies of the WLQ^(10,20)^.

In addition to analyzing the psychometric properties of the complete factorial model of the WLQ, the reduced versions of the instrument (WLQ-16 and WLQ-8) for the sample were tested. It was verified that the eight-item model did not fit to the data and that WLQ-16, although it did show adjustment, was not superior to the adjustment achieved with the 25-item version of the WLQ for the sample. Thus, for our sample, the complete version of the WLQ represented the factorial model that best fit to the sample. This fact suggests the importance of using complete versions of psychometric instruments during validation processes, given the intrinsic relationship between the cultural context and the characteristics of the sample for adjusting the factor models^(32)^.

Regarding the scores of the WLQ domains, the results showed that the demands related to productivity, the time to perform the tasks inherent to the function performed, and the psychological and mental loads required by work in hospital institutions were the workloads that most collaborated for the occurrence of presenteeism among nursing workers. In this sense, it is highlighted that presenteeism among nursing workers is directly related to the stressors of the work environment, which can interfere with the quality of life and well-being of these individuals^(42)^.

The analysis of the correlation between the demographic variables and the WLQ domains showed that there were no statistically significant differences for the occurrence of presenteeism between women and men and/or among the nurses, nursing technicians and assistants in the sample. It was also observed that individuals with less working time have a higher weekly workload in hospitals, and are therefore more susceptible to presenteeism.

The analysis of the correlations between the domains of the WLQ and the working time and workload variables showed that the longer the working time in the institution, the greater the mental demands and those related to productivity at work, which indicates a greater risk of psychological illness among nursing workers throughout their time in the profession. In addition to that, these correlations indicated that nurses, nursing technicians and assistants who have been working at the hospital for less time have a higher workload and experience greater physical demands at work, being more prone to the risk of physical illness.

These results are reinforced by the data obtained related to the illness profile of the participants, who reported being mainly affected by musculoskeletal diseases (especially low back pain), anxiety, depression and respiratory problems, and reflect the reality of the work experienced by nursing professionals in the hospital institutions under study.

Studies on the working conditions in Brazilian hospitals have shown that nursing professionals face stressful situations related to patient care and work organization on a daily basis, such as low staffing, pressure from management, and lack of individual appreciation, among other factors, which generates physical and mental overload for these workers, reduces their quality of life at work and contributes to their illness^(3-4,44)^.

One of the ways to evaluate presenteeism is to measure its consequences, such as the loss of productivity at work, obtained in this study by estimating the WLQ loss of productivity index. For the nursing workers under study, this index was approximately 20%, which means that approximately one fifth of the productivity of these professionals is lost due to problems that affect the physical and mental health of these individuals and that such troubles are not properly treated, causing negative impacts not only on the workers’ health, but also on the quality of care provided to the patients in hospital institutions.

As limitations of this study, it is highlighted that the cross-sectional design and non-probabilistic sampling method, carried out with a specific sample of workers, do not allow for the establishment of causal relationships and for the generalization of the results. Another limiting factor was the sample size used, which was not sufficient to test the invariance of the presenteeism measurement instrument, which could be interesting for assessing the external validity of the data.

## Conclusion

The results obtained in this research showed that the Work Limitations Questionnaire represents a valid and reliable instrument for assessing presenteeism in nursing workers. Furthermore, it is considered that this study has the contribution of offering an instrument that can be used as a tool for assessing the loss of productivity due to health problems among nursing workers, which can subsidize decisions by health service managers and coordinators aimed at implementing health promotion programs at work.
